# Effectiveness of electrical stimulation combined with pelvic floor muscle training on postpartum urinary incontinence

**DOI:** 10.1097/MD.0000000000014762

**Published:** 2019-03-08

**Authors:** Xiao-xing Ma, An Liu

**Affiliations:** a Department of Gynecology; b Department of Urology, Yan’an People's Hospital, Yan’an, China.

**Keywords:** effectiveness, electrical stimulation, pelvic floor muscle training, postpartum urinary incontinence, systematic review

## Abstract

**Background:**

Previous clinical trials have reported that electrical stimulation (ES) combined with pelvic floor muscle training (PFMT) can be used to treat postpartum urinary incontinence (PPUI) effectively. However, no systematic review has investigated the effectiveness and safety of ES plus PFMT for the treatment of patients with PPUI. In this systematic review, we will firstly assess the effectiveness and safety of ES and PFMT for treating PPUI.

**Methods:**

In this study, we will search the following electronic databases: Cochrane Library, Web of Science, Springer, MEDLINE, EMBASE, Cumulative Index to Nursing and Allied Health Literature, Allied and Complementary Medicine Database, Chinese Biomedical Literature Database, and China National Knowledge Infrastructure from inceptions to the present without language restrictions. All eligible randomized controlled trials (RCTs) on the effectiveness of ES plus PFMT for PPUI will be included. We will also search grey literature to avoid missing any other potential qualified studies. Two authors will independently conduct the study selection, data extraction, and risk of bias assessment. A third author will be consulted to solve any disagreements between 2 authors. RevMan 5.3 Software will be used to pool the data and to carry out the meta-analysis.

**Results:**

This study will provide high quality evidence of ES and PFMT for PPUI. The primary outcome includes symptoms improvement. The secondary outcomes consist of incontinence-specific quality of life, clinician's observations, and adverse effects.

**Conclusion:**

The findings of this study will summary up-to-dated evidence for judging whether ES combined PFMT is an effective intervention for PPUI or not.

**Ethics and Dissemination:**

This study does not needs ethical approval, because it will not involve individual patient data. Its findings will be disseminated through peer-reviewed journals.

**Systematic review registration:**

CRD42019122540.

## 1 Introduction

Urinary incontinence (UI) is a common disorder during the period of pregnancy-puerperium cycle.^[1–3]^ It has been reported that the prevalence of this condition ranges from 18.6% to 75% during the period of pregnancy, and 6% to 31% during the period of postpartum.^[4–6]^ Several factors account for the UI during the period of pregnancy and the puerperium, such as pregnancy itself, hormonal changes, anatomical injury after birth, and dynamic forces.^[7–9]^

UI occurred during the postpartum period also called postpartum urinary incontinence (PPUI). It can greatly lead to the decreased quality of life during the puerperal period.^[10–12]^ Several managements are reported to treat this condition, such as acupuncture, electrical stimulation (ES), pelvic floor muscle training (PFMT), and the combination of ES and PFMT effectively, especially for ES plus PFMT.^[13–23]^ Although 2 previous systematic reviews addressed the effectiveness of ES for UI in women,^[24,25]^ no systematic review specifically investigated the effectiveness and safety of ES plus PFMT for the treatment of patients with PPUI. Thus, this systematic review will specifically focus on assessing the effectiveness and safety of ES combined with PFMT for patients with PPUI.

## 2 Methods and analysis

### 2.1 Study registration

The systematic review protocol has designed according to the Preferred Reporting Items for Systematic Reviews and Meta-Analysis Protocol statement guidelines,^[26]^ and has been registered on PROSPERO (CRD42019122540).

### 2.2 Inclusion criteria for study selection

#### 2.2.1 Types of studies

Only randomized controlled trials (RCTs) of ES combined PFMT for the treatment of PPUI will be considered for inclusion. However, non-RCTs, non-controlled studies, and non-clinical studies will not be considered for inclusion.

#### 2.2.2 Types of participants

Participants with a clinically confirmed diagnosis of PPUI will be included regardless their age, sex, race, education, or economic status.

#### 2.2.3 Types of interventions

##### 2.2.3.1 Experimental interventions

Any forms of ES plus PFMT in the experimental group will be considered for inclusion. However, the studies with ES, or PFMT alone, or other therapies combined with one of them, or both will not be included.

##### 2.2.3.2 Control interventions

Any treatments, except ES, PFMT, or ES combined with PFMT in the control group will be included.

#### 2.2.4 Type of outcome measurements

##### 2.2.4.1 Primary outcomes

The primary outcome includes symptoms improvement. It can be measured by self-report of UI, voiding diary, and any other measurement tools.

##### 2.2.4.2 Secondary outcomes

The secondary outcomes include incontinence-specific quality of life, as assessed by Incontinence Quality of Life Questionnaire, or any others; clinician's observations, including the observation of UI; urodynamic measurements; and pelvic floor muscle function and strength. In addition, any adverse effects will also be assessed.

### 2.3 Literature search

We will search the following electronic databases: Cochrane Library, Web of Science, Springer, MEDLINE, EMBASE, Cumulative Index to Nursing and Allied Health Literature, Allied and Complementary Medicine Database, Chinese Biomedical Literature Database, and China National Knowledge Infrastructure from their inceptions to the present by using predefined detailed search strategy for each database. All the databases will be searched without language limitations. We have provided the sample search strategy for Cochrane Library in Table 1. The identical search strategy will also be applied to other electronic databases. In addition, grey literature, such as clinical trial registry, reference lists of included studies, and conference proceedings will also be searched.

**Table 1:**
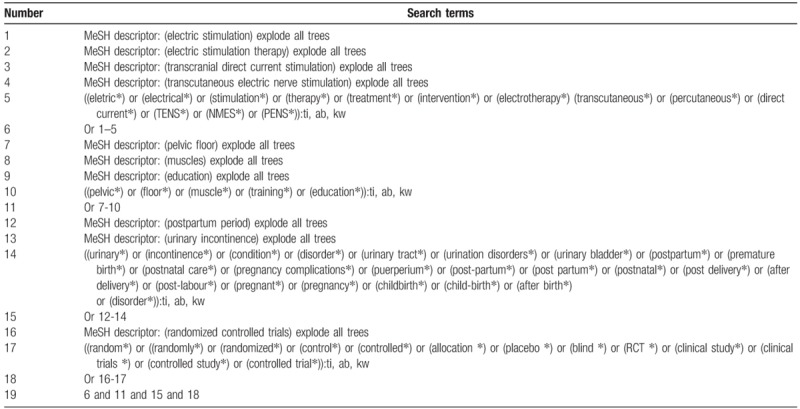
Search strategy applied in Cochrane Library database.

### 2.4 Data collection and management

#### 2.4.1 Study selection

Two authors will independently conduct the study selection by scanning titles or abstracts, and reading full texts if the decision cannot be judged through the insufficient information from titles and abstracts. All the procedures of study selection will be carried out according to the predefined eligibility criteria. A third author will be invited to solve any divergences between 2 authors. The study selection procedure will follow and be documented in the Preferred Reporting Items for Systematic Reviews and Meta-Analyses flow chart.

#### 2.4.2 Data extraction and management

Two authors will utilize predefined standardized form to extract the data independently. The extraction sheet includes study characteristics, such as title, first author, year of publication, country; patient characteristics, including age, race, sex, number of patients; study methods, comprising of randomization, blinding, concealment, and any other risk of bias information; interventions, such as dosage, frequency, duration of each group; and outcomes, consisting of primary, secondary outcomes, and adverse effects. If any disagreements regarding the data extraction happened between 2 authors, a third author will be consulted by discussion. Additionally, if any insufficient information or missing data are identified duration the period of data extraction, the primary authors will be contacted to inquire those data. If those data are not achievable, we will only pool and analyze the available data.

### 2.5 Risk of bias assessment

The methodological quality for each qualified study will be assessed by using Cochrane risk of bias tool. It comprises of 7 domains, and each field is further classified as high risk of bias, unclear risk of bias, or low risk of bias. Two authors will independently carry out the methodological assessment for each study. Any disagreements will be solved by discussion with a third author.

### 2.6 Statistical analysis

RevMan 5.3 software (Cochrane Community, London, UK) will be used to pool the data and to conduct the meta-analysis if it is possible. All dichotomous data will be expressed as risk ratio and 95% confidence intervals (CIs). All continuous data will be presented as mean difference or standardized mean difference and 95% CIs. *I*^2^ test will be used to detect the heterogeneity among included studies. Acceptable heterogeneity will be considered if *I*^2^ is <50%, and data will be pooled by using fixed-effect model. On the other hand, substantial heterogeneity will be considered if *I*^2^ is >50%, and data will be pooled by using random-effect model. Under such situation, we will carry out subgroup analysis in accordance with different locations, treatments, and outcome instruments. If significant heterogeneity is still identified after the subgroup analysis, we will not pool the data, and will not perform meta-analysis. Instead, we will just report a narrative summary. In addition, sensitivity analysis will also be considered to perform by eliminating the impact of low quality studies. Furthermore, if >10 eligible RCTs are included, we will also conduct the funnel plots, and Eggers Regression test.^[27,28]^

## 3 Discussion

PPUI is very frequent condition during the puerperal period. Clinical studies reported that ES combined PFMT can treat PPUI effectively,^[18–23]^ and has widely used in the clinical practice. However, no systematic review regarding the effectiveness and safety of ES plus PFMT has been conducted for treating PPUI so far. Thus, this high quality systematic review will firstly evaluate the effectiveness and safety of ES plus PFMT for the treatment of PPUI. The results of this study will pool the outcome data and will also provide the current evidence on the effectiveness and safety of ES plus PFMT for the treatment of PPUI. This review may present solid data and robust evidence to the clinicians, researchers, and health policy makers.

## Author contributions

**Conceptualization:** Xiao-xing Ma, An Liu.

**Data curation:** Xiao-xing Ma, An Liu.

**Formal analysis:** Xiao-xing Ma, An Liu.

**Funding acquisition:** An Liu.

**Investigation:** An Liu.

**Methodology:** Xiao-xing Ma, An Liu.

**Project administration:** An Liu.

**Resources:** Xiao-xing Ma.

**Software:** Xiao-xing Ma.

**Supervision:** An Liu.

**Validation:** Xiao-xing Ma, An Liu.

**Visualization:** Xiao-xing Ma, An Liu.

**Writing - original draft:** Xiao-xing Ma, An Liu.

**Writing - review & editing:** Xiao-xing Ma, An Liu.
